# Identification and independent validation of a stable yield and thousand grain weight QTL on chromosome 6A of hexaploid wheat (*Triticum aestivum L*.)

**DOI:** 10.1186/s12870-014-0191-9

**Published:** 2014-07-18

**Authors:** James Simmonds, Peter Scott, Michelle Leverington-Waite, Adrian S Turner, Jemima Brinton, Viktor Korzun, John Snape, Cristobal Uauy

**Affiliations:** 1 John Innes Centre, Norwich Research Park, Norwich NR4 7UH, UK; 2 KWS Lochow GMBH, Ferdinand-von-Lochow-Str. 5, Bergen-Wohlde 29303, Germany; 3 National Institute of Agricultural Botany, Huntingdon Road, Cambridge CB3 0LE, UK

**Keywords:** Wheat, Yield, Grain size, Grain shape, Green canopy duration, QTL, NILs

## Abstract

**Background:**

Grain yield in wheat is a polygenic trait that is influenced by environmental and genetic interactions at all stages of the plant’s growth. Yield is usually broken down into three components; number of spikes per area, grain number per spike, and grain weight (TGW). In polyploid wheat, studies have identified quantitative trait loci (QTL) which affect TGW, yet few have been validated and fine-mapped using independent germplasm, thereby having limited impact in breeding.

**Results:**

In this study we identified a major QTL for TGW, yield and green canopy duration on wheat chromosome 6A of the Spark x Rialto population, across 12 North European environments. Using independent germplasm in the form of BC_2_ and BC_4_ near isogenic lines (NILs), we validated the three QTL effects across environments. In four of the five experiments the Rialto 6A introgression gave significant improvements in yield (5.5%) and TGW (5.1%), with morphometric measurements showing that the increased grain weight was a result of wider grains. The extended green canopy duration associated with the high yielding/TGW Rialto allele was comprised of two independent effects; earlier flowering and delayed final maturity, and was expressed stably across the five environments. The wheat homologue (*TaGW2*) of a rice gene associated with increased TGW and grain width was mapped within the QTL interval. However, no polymorphisms were identified in the coding sequence between the parents.

**Conclusion:**

The discovery and validation through near-isogenic lines of robust QTL which affect yield, green canopy duration, thousand grain weight, and grain width on chromosome 6A of hexaploid wheat provide an important first step to advance our understanding of the genetic mechanisms regulating the complex processes governing grain size and yield in polyploid wheat.

## Background

Wheat (*Triticum aestivum* L.) is one the world’s major staple crops, supplying approximately twenty percent of the global total calorie intake [[1]]. There have been considerable advances in yield since the introduction of the ‘green revolution’ genes. However for the UK, Europe and other countries worldwide, the last decade has seen a decline in the rate of genetic gains, with yield plateaus in some environments [[2],[3]]. Furthermore, with the global demand for wheat rising faster than the rate of yield improvement, there is a genuine threat to food security. Therefore the discovery, understanding and eventual incorporation of genes and alleles that beneficially influence yield are major targets for breeding programs worldwide.

The grain yield of wheat and cereals in general, is a polygenic and highly complex trait that is influenced by environmental and genetic interactions at all stages of the plant’s growth [[4]]. To facilitate its study, yield is usually broken down into three main components; number of spikes per surface area, grain number per spike, and thousand grain weight (TGW). These yield components are sequentially fixed during the growth cycle, vary in terms of their heritability, and are not always positively correlated with yield [[5]]. TGW is usually stably inherited [[6]] and can be further broken down into individual components including physical parameters (grain length, width, area) and grain filling characteristics, which are also under independent genetic control [[7]]. These include both the rate and duration of the grain filling process [[8]], the latter being normally phenotyped as green canopy duration after heading [[9]].

In the past decade, there have been significant advances in our understanding of the genetic control of grain size, shape, and grain filling parameters in the diploid crop species rice (*Oryza sativa*; reviewed in [[10],[11]]). Several genes with relatively large effects have been identified through map-based cloning and support the independent genetic control of grain length, width and grain filling parameters. This differs from our limited understanding in polyploid wheat where several studies have identified quantitative trait loci (QTL) for grain size and shape [[12]–[15]], but no gene has yet been cloned. Moreover, many of these QTL are in relatively wide genomic regions and have not been validated and fine-mapped using independent germplasm, therefore having limited impact in breeding.

In rice, *OsGW2* encodes a previously unknown RING-type E3 ubiquitin ligase and functions as a negative regulator of grain width and weight [[16]]. Recently, several studies have examined the role of the wheat homologue (*TaGW2*) on grain size parameters, although contradictory results have been reported. Two studies have described a SNP at position −593 upstream of the putative start codon as significantly associated with wider grains and increased TGW in Chinese germplasm [[17],[18]]. However, the results are directly contradictory since each study found the positive association with the opposite SNP at the exact −593 position. Despite the alternative alleles at this site, both studies identify a negative association between *TaGW2* expression levels and grain width. Yang et al. [[19]] identified a *TaGW2* frame-shift mutation in a large-kernel variety, and associated this mutant allele with increased grain width and TGW in a large F_2:3_ population. This mutant mimics the original rice *OsGW2* truncation allele [[16]], suggesting that *TaGW2* and *OsGW2* share a conserved mechanism (negative regulation of grain size). However, down-regulation of *TaGW2* through RNA interference (RNAi) resulted in decreased grain size and TGW in wheat [[20]], suggesting that *TaGW2* is a positive regulator of grain size with a divergent function to that of rice *OsGW2*. Taken together, it is difficult to conclude the exact effect of *TaGW2* on grain size and TGW in wheat due to the discrepant studies published to date.

The objective of this study was to evaluate a doubled haploid mapping population across North European environments for thousand grain weight, yield and additional morphological and developmental traits. We identified a meta-QTL for TGW, yield, and green canopy duration on wheat chromosome 6A. We developed near isogenic lines (NILs) to validate the 6A QTL effects across environments, and morphometric grain analyses were conducted to determine the specific grain size components being affected by the QTL.

## Results

### Genetic map and QTL analyses

**Figure 1:**
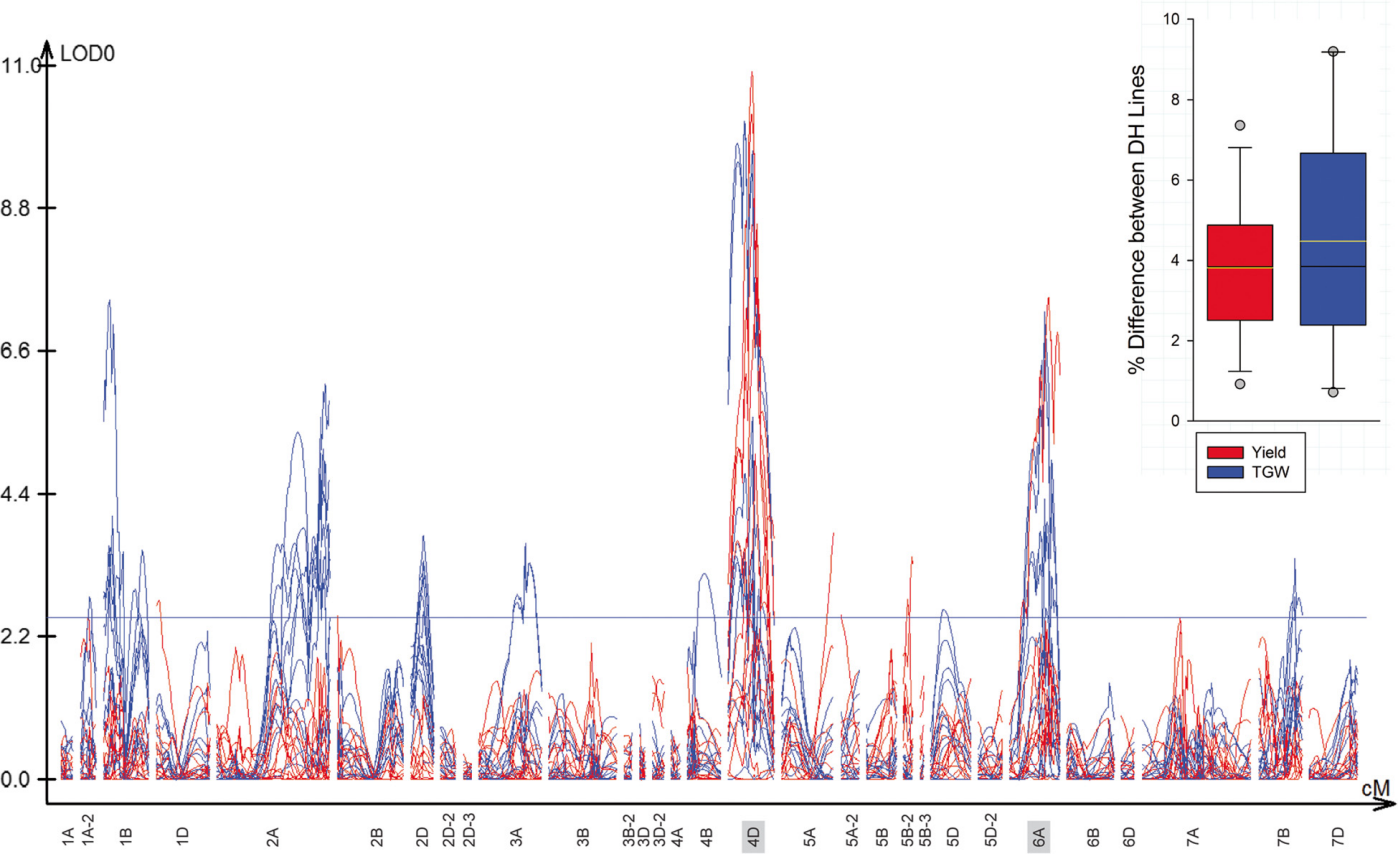
**QTL analysis for yield and thousand grain weight.** Genome-wide QTL analyses for yield (red) and thousand grain weight (blue) in the Spark x Rialto DH population, across five environments (Norwich UK, Sandringham UK, Scotland, France, Germany) and in three years (2001, 2002 and 2003). The threshold value for significance is set at 2.5 LOD. Consistent, significant effects for both yield and thousand grain weight were observed on chromosomes 4D (*Rht-D1*) and 6A. Inset: the box and whisker plot exhibits the percentage difference between DH lines fixed for the QTL region on chromosome 6A, with the median and mean denoted by the black and yellow line, respectively.

The Spark x Rialto DH linkage map was developed using 263 markers, including 170 SSRs, 89 DArT, 2 protein markers, the *Rht-D1b* perfect marker, and a morphological GA test. The total length of the map was 1,471 cM across 30 linkage groups which were assigned to specific chromosomes using published consensus maps [[21]]. Seven chromosomes included at least two linkage groups (1A, 2D, 3B, 3D, 5A, 5B, 5D) which were considered separately for the QTL analyses. Across locations, Rialto had significantly (*P* < 0.001) larger thousand grain weight (49.1) than Spark (40.9) and this was consistent across years (*P* = 0.49; interaction Parent*Year). In the DH population, five QTL for grain size were consistently identified across at least five locations on chromosomes 1B, 2A, 2D, 4D and 6A (Figure 1, Additional file 1). The Rialto allele conferred the increased grain size for four QTL (1B, 2A, 2D and 6A), whereas Spark provided the increased grain size on chromosome 4D.

Two QTL for yield were identified across environments on chromosomes 4D and 6A, and both co-localized with the previously identified thousand grain weight QTL (Figure 1, Additional file 1). The 4D yield and TGW QTL peaks coincide with the *Rht-D1* dwarfing gene whose pleiotropic effect on yield and yield components has been comprehensively documented [[22],[23]]. The yield and TGW QTL on chromosome 6A were detected between markers *Xgdm36* and *Xgwm570* with Rialto providing the increasing allele for both traits. This chromosome region was the only one apart from *Rht-D1* that significantly influenced both yield and grain weight in the Spark × Rialto DH population. Therefore, the grain size and yield QTL on chromosome 6A were selected for further study and designated as *Qtgw-jic.6A* and *Qyld-jic.6A*, respectively.

A factorial ANOVA was conducted including environment and all two-way interactions to assess individual QTL effects and epistatic interactions between *Rht-D1* and *Qtgw-jic.6A/Qyld-jic.6A*. There was a significant effect of *Rht-D1* and the 6A QTL on TGW (*P* < 0.001) and yield (*P* < 0.001) and strong interactions between QTL and environment for both traits (*P* < 0.01). However, there was no significant genetic interaction between *Rht-D1* and *Qtgw-jic.6A/Qyld-jic.6A* (*P* = 0.13 and *P* = 0.17, respectively).

DH lines homozygous for the 6A Rialto region between *Xgdm36* and *Xgwm570* had a significant increase in yield of 3.82 ± 0.5% across environments compared to DH fixed for the Spark allele (*P* < 0.001, Figure 1 inset, Additional file 2). These gains ranged from 0.9% (Germany 2002) to 7.4% (Scotland 2003). Increases in TGW were more variable, averaging 4.47 ± 0.8% and ranging from 0.7% (France 2002) to 9.2% (Germany 2003) (Figure 1 inset, Additional file 2). The mean yield and TGW for the selected DH lines at each environment were positively correlated (*r* = 0.51), although this was not significant (*P* = 0.09) due to differential effects across environments. For example, DH lines fixed for the *Qyld-jic.6A* Rialto segment exhibited yield improvements of 7.4% in Scotland (2003), however TGW was only increased by 3.5% at this site (*r* = 0.09; *P* = 0.42), whereas in other environments (Church Farm 2001 and 2002, Sandringham 2003, Germany 2002, France 2003) TGW and yield were significantly correlated (*r* > 0.23; *P* < 0.05). These results suggest that TGW is an important yield component underlying the *Qyld-jic.6A* effect, however, the relative contribution of increased TGW on yield varied across environments.

In addition to the TGW and yield effects, a QTL for green canopy duration after heading (time from heading to canopy senescence) was also identified between *Xgdm36* and *Xgwm570* and designated *Qgcd-jic.6A*. Similar to *Qtgw-jic.6A* and *Qyld-jic.6A*, the Rialto allele had the positive effect extending green canopy duration significantly by 2.0 ± 0.3 days (*P* < 0.001; ranging from 1.2 to 2.5 days) compared to the Spark allele, across all four environments analysed. Green canopy duration showed a significant (*P* = 0.04) negative correlation with yield across the four locations (*r* = −0.12), although the correlations were not significant in three of the four environments. There was no significant correlation (*P* = 0.42) between green canopy duration and TGW (*r* = −0.04) across environments. Chromosome 6A had no effect on plant biomass, harvest index, seeds/spike, and seeds/spikelet across locations.

### Multi-trait multi-environment QTL analyses

**Figure 2:**
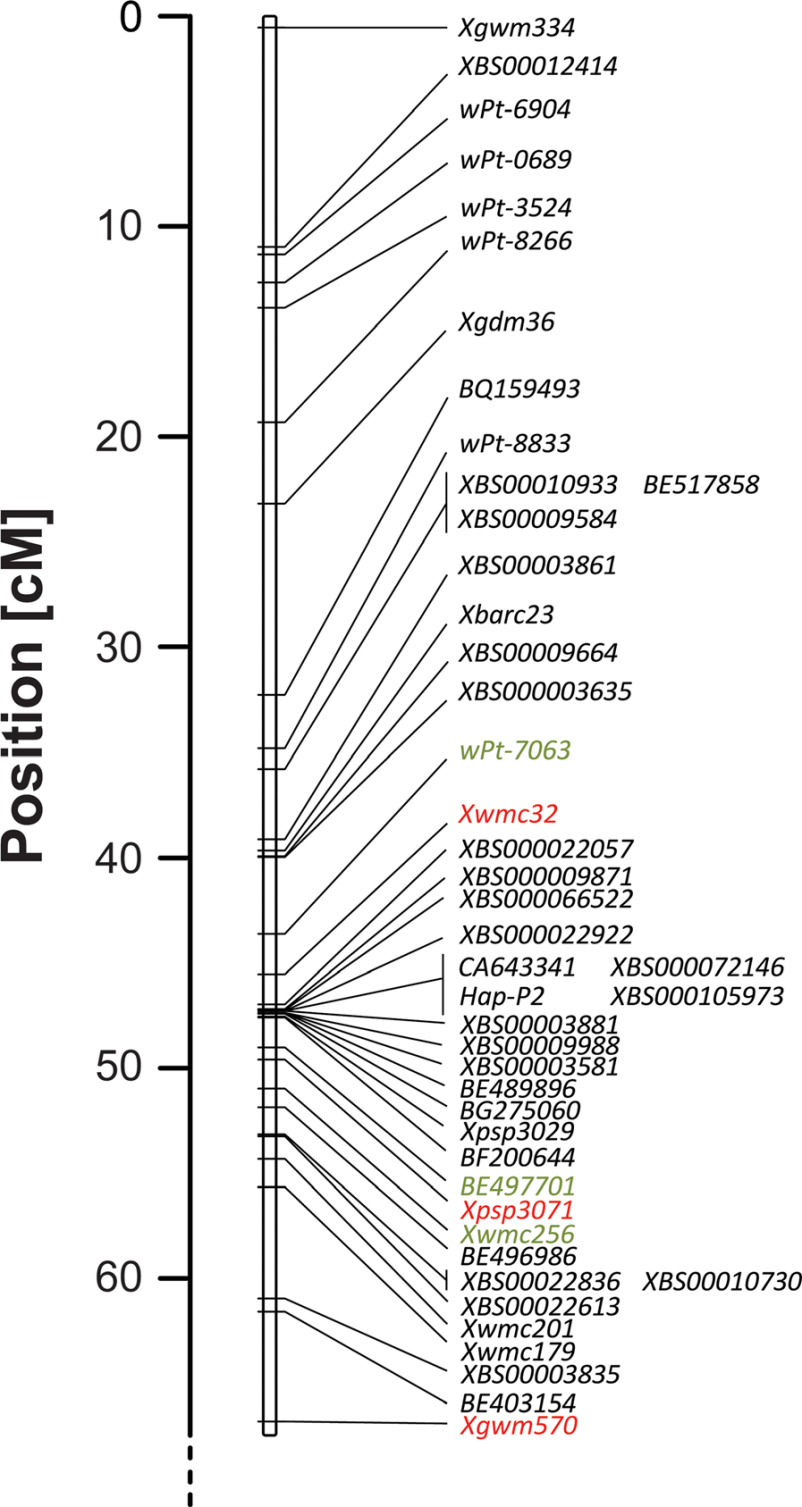
**Genetic map of chromosome 6A for the Spark x Rialto DH population.** Markers coloured green correspond to the marker with the highest LOD score for yield (*wPt-7063*), TGW (*BE497701*) and Green Canopy Duration after heading (*Xwmc256*) from MTME analysis. Markers coloured red represent those used for marker assisted selection during the development of Near Isogenic Lines.

The marker resolution across chromosome 6A was increased by the addition of 19 SNP-based markers (Figure 2). The improved 6A genetic map covers a genetic distance of 66 cM, ranging from *Xgwm334* at the distal end of the short arm, to *Xgwm570* which maps mid-way along the long arm (bin map location 6AL8-0.90-1.00 [[24]]).

**Figure 3:**
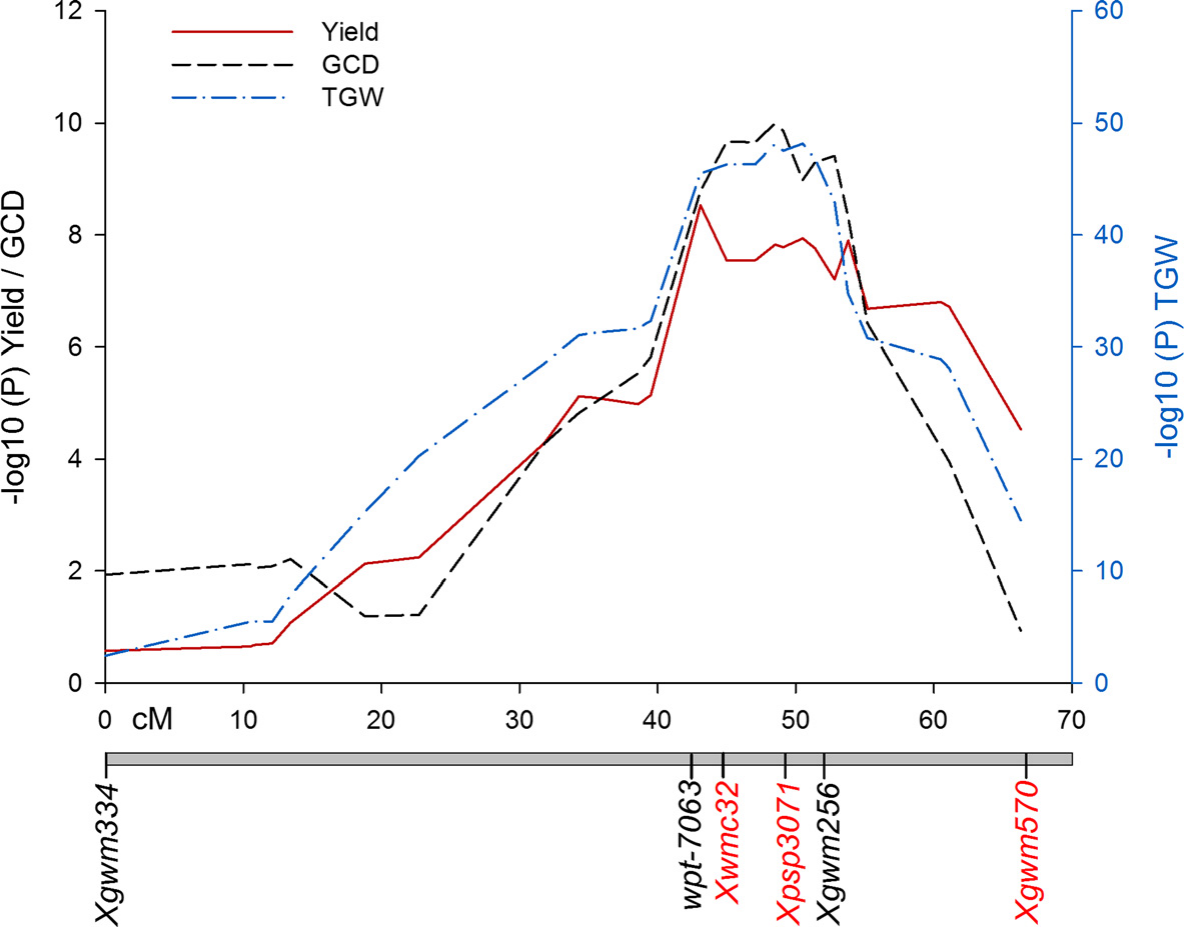
**Multi Environment QTL Mapping.** Co-localisation of QTL for yield (red solid line), thousand grain weight (blue dash line), and green canopy duration (black dash line) on chromosome 6A across multiple environments and years. Flanking markers used for selection during the production of Near Isogenic Lines are indicated in red. Genetic distances (cM) correspond to those shown in Figure 2.

Using the improved genetic map for chromosome 6A, the original phenotypic data was reanalysed using Multi-Trait Multi-Environment (MTME) analysis for a more precise positioning of the QTL across all environments [[25]]. For yield, *Qyld-jic.6A* was identified as significant across the interval from *Xgdm36* (22.7 cM) to *Xgwm570* (66.3 cM), with the peak at *wPt-7063* (43.1 cM) (Figure 3). Significant markers within this region were identified in 9 of the 12 environments, all with Rialto as the beneficial allele (Additional file 3). For *Qtgw-jic.6A* the QTL encompasses the whole linkage group with all markers showing significance and with Rialto providing the positive allele in all cases. The QTL reaches its highest significance between *wPt-7063* (43.1 cM) to *Xgwm256* (51 cM), with the peak at *BE497701* (47 cM). Significant markers were observed in all environments apart from France and Germany in 2002 (Additional file 3). *Qgcd-jic.6A* spans the region from *BE517858* (32 cM) to *BE403154* (58.8 cM), has its highest significance at *Xgwm256* (51 cM) and was significant across all four environments (Additional file 3). The MTME analysis established that the yield, TGW and green canopy duration effects are all co-localised to a 8 cM region between *wPt-7063* (43.1 cM) to *Xgwm256* (51 cM) and that these effects are stable across different North European environments. In addition to these major effects, a minor QTL for tiller number was identified at *BQ159493* (31.8 cM) in three of the five environments (Additional file 3), mapping distal to the location of *Qyld-jic.6A*, *Qtgw-jic.6A* and *Qgcd-jic.6A*. In this case, however, Spark provided the positive effect allele.

### Validation of *Qtgw-jic.6A* and *Qyld-jic.6A* using near isogenic lines (NILs)

To independently validate these multiple effects, BC_2_ and BC_4_ NILs segregating for the QTL segment (*BQ195493* to *Xgwm570*) were developed. NILs were assessed for yield, TGW and grain size parameters in five environments, four in England (2010–2013) and one in Germany (2012G) (Table 1). Overall, the Rialto 6A NIL significantly increased yield (*P* < 0.001), although there was a strong interaction with environment (*P* < 0.001). In four of the five experiments yield increases were observed from the Rialto NILs (ranging from 3.2% to 9.8% per plot), with three of these effects being significant and one non-significant (*P* = 0.08; 2011). However, a significant decrease in plot yield was observed in 2012 in England, where Rialto NILs had 6.3% lower yield (*P* < 0.001). For TGW, a significant increase was observed in Rialto NILs across locations (*P* < 0.001), although again there was a significant interaction with environment (*P* < 0.001) (Table 1). Similar to plot yield, Rialto increased TGW in four environments (ranging from 2.3% to 8.8%) with three being significant and one borderline non-significant (*P* = 0.06, 2010). No effects on TGW were observed in 2012. The plot yield and TGW of the 6A NILs was positively correlated (*r* = 0.21, *P* < 0.001) across environments. Similar to the DH population, no consistent differences were observed between NILs for plant biomass, harvest index, spike length, spikelet number, spike yield, seeds/spike, and seeds/spikelet (Additional file 4).

**Table 1:** **Yield and thousand grain weight of the 6A BC**_**2**_**(2010–2012) and BC**_**4**_**(2013) NILs**

	**Yield (kg/plot)**					**Thousand grain weight (g)**				
NIL Allele	2010	2011	2012	2012G	2013	2010	2011	2012	2012G	2013
Spark	4.205	2.904	3.802	2.930	5.336	35.0	48.5	38.6	37.0	35.1
Rialto	4.393	2.995	3.561	3.217	5.571	35.8	49.7	38.4	39.5	38.2
delta	4.5%^**^	3.2%^NS^	−6.3%^***^	9.8%^***^	4.4%^*^	2.3%^NS^	2.3%^***^	−0.5%^NS^	6.9%^***^	8.8%^***^

Performance of NILs with either the recurrent parent (Spark) or the introgressed region (Rialto) across 5 environments. Significant differences are represented by * (*P* <0.05), ** (*P* <0.01) and *** (*P* <0.001); non-significant effects are also indicated (NS). Delta refers to the difference between the Rialto and Spark NIL phenotypes as a percentage of the Spark NIL.

**Figure 4:**
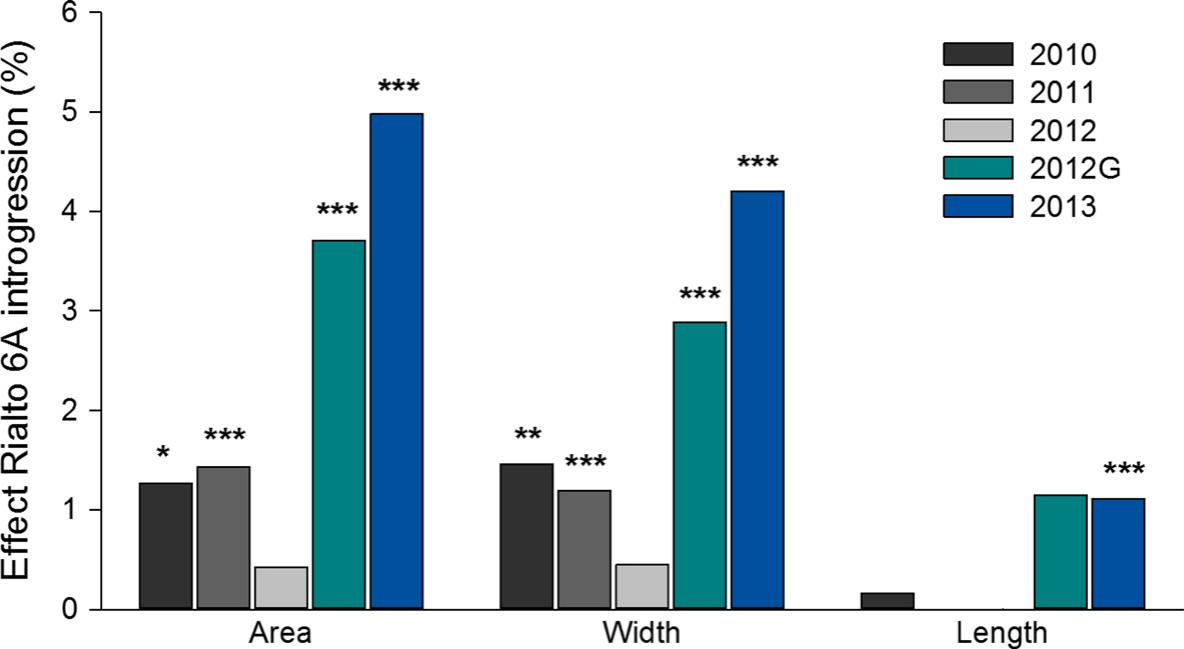
**Effect of 6A introgression on grain morphometric parameters.** Percentage increase conferred by the Rialto introgression in the 6A BC_2_ NILs (2010–2012) and BC_4_ NILs (2013). Significant differences are represented by * (*P* <0.05), ** (*P* <0.01) and *** (*P* <0.001).

**Figure 5:**
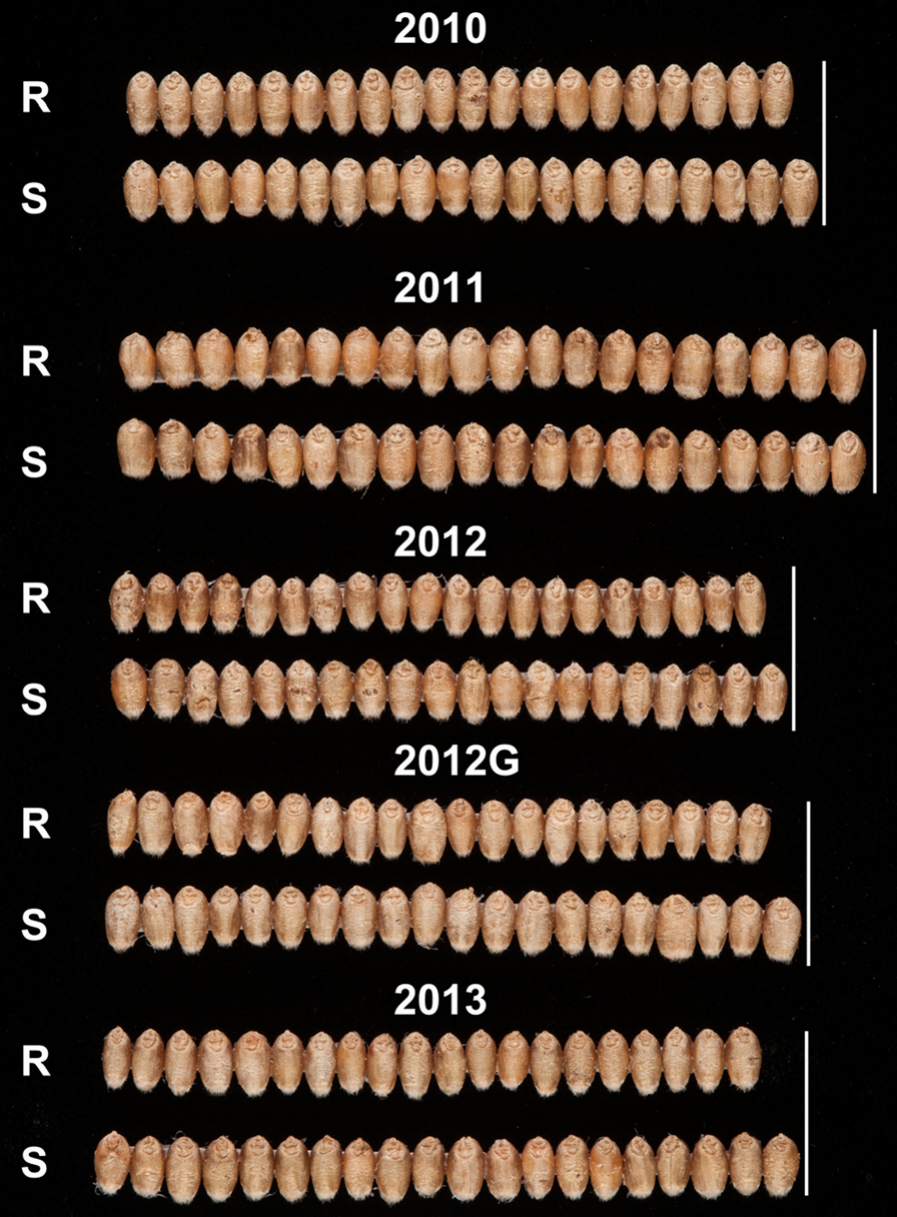
**Field grown wheat grains across environments.** Twenty representative grains from selected BC_2_ NILs (2010–2012) and BC4 NILs (2013) carrying the Spark (S) or Rialto (R) introgression showing differences in grain width between NIL pairs and across environments.

Morphometric measurements of the grain were analysed to assess the source of the increases in TGW (Figure 4). Total grain area was significantly increased in four of the five environments in an equivalent degree to the TGW results. This was expected based on the high positive correlation between these two measures (*r* = 0.86; *P* < 0.001). The increased grain size and weight was due primarily to significantly wider grains in the Rialto NILs. Grains from Rialto NILs were on average 2.0% wider than grains from Spark NILs (ranging from 0.4% to 4.2%) and this was highly significant in four of the five environments tested. No significant effect in grain length was observed between NILs across locations with the exception of 2013 where Rialto NILs had significantly longer grains than Spark NILs (1.1%, *P* < 0.01). The alignment of twenty grains of equivalent BC lines illustrates the difference in grain width between BC_2_ and BC_4_ NILs and the variation in grain size across environments (Figure 5).

### Validation of developmental traits using NILs

Developmental characteristics including heading date, physiological maturity and the calculation of green canopy duration were assessed in the UK environment over four years using BC_2_ (2010–2012) and BC_4_ (2013) NILs. Despite extreme variation in growing conditions over this period, robust significant effects were observed for all three traits (Table 2). For heading date, NILs containing the Rialto introgression flowered earlier by 0.89 ± 0.06 days (*P* < 0.001; ranging from 0.73 to 1 days). Rialto NILs also were significantly later at reaching physiological maturity compared to Spark NILs in all four seasons by 1.59 ± 0.4 days (*P* < 0.001; ranging from 0.73 to 2.58 days). The combination of these two effects significantly lengthens the green canopy duration for the Rialto NILs by an average of 2.48 ± 0.37 days across environments (*P* < 0.001; ranging from 1.73 to 3.44 days). The non-significant interaction (*P* = 0.18) between green canopy duration and environment highlights the consistent effect of the Rialto allele across growing seasons, despite the large variation in average green canopy duration between years (ranging from 44 days in 2010 to 75 days in 2011). These results suggest that the original green canopy duration QTL *Qgcd-jic.6A* is in fact a combination of two distinct effects, earlier flowering and delayed final maturity, which both co-localize to the 6A region and are stably expressed across environments.

**Table 2:** **Developmental traits of the BC**_**2**_**(2010–2012) and BC**_**4**_**(2013) NILs**

	**Heading (Days)**				**Maturity (Days)**				**Green canopy duration (Days)**				**Tiller no. (1 m row)**		
NIL Allele	2010	2011	2012	2013	2010	2011	2012	2013	2010	2011	2012	2013	2011	2012	2013
Spark	238.5	226.1	256.4	256.6	282.0	299.5	322.8	301.6	43.5	73.4	66.5	45.1	76.9	174.7	97.6
Rialto	237.5	225.4	255.5	255.6	282.7	301.3	325.4	302.8	45.2	76.0	69.9	47.2	76.1	167.5	92.2
delta	−1.00^**^	−0.73^***^	−0.86^**^	−0.95^***^	0.73^**^	1.86^***^	2.58^***^	1.20^***^	1.73^***^	2.59^***^	3.44^***^	2.15^***^	−0.8^NS^	−7.2^*^	−5.4^**^

Heading and maturity are calculated as the number of days from drilling, whereas green canopy duration is determined by the difference between heading and maturity dates. Significant differences are represented by *(*P* <0.05), **(*P* <0.01) and ***(*P* <0.001); non-significant effects are also indicated (NS). Delta refers to the difference between the Rialto and Spark NIL phenotypes in absolute values.

**Figure 6:**
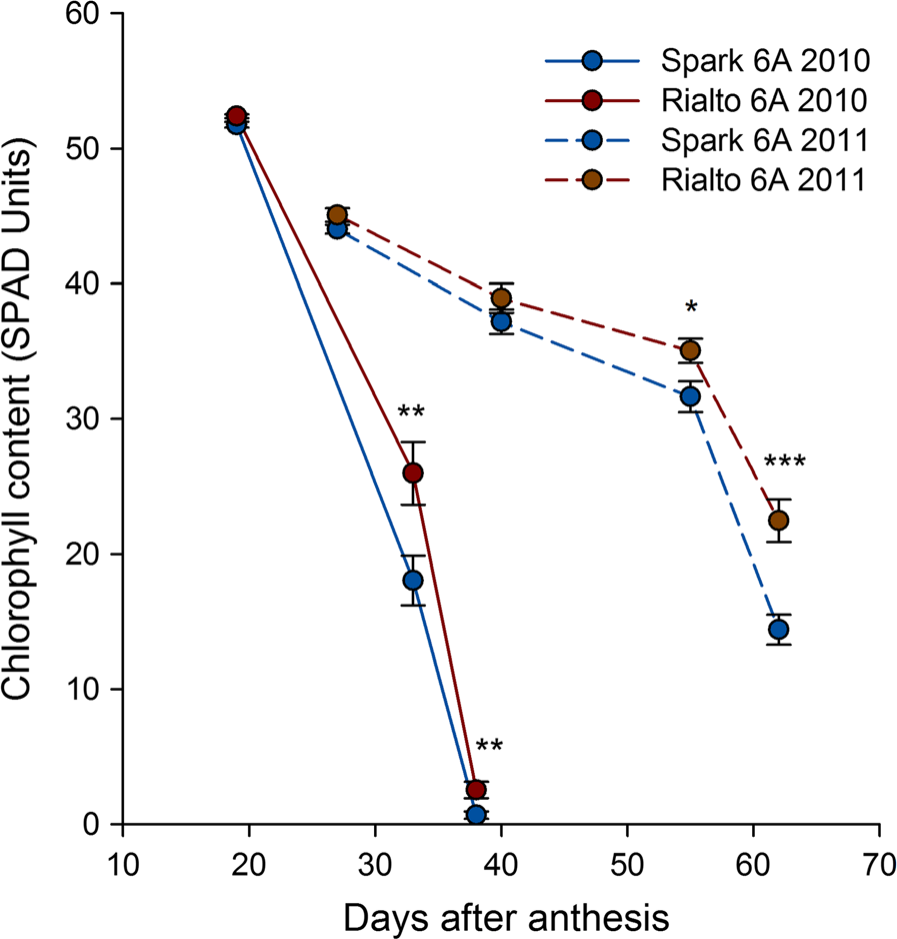
**Relative chlorophyll content (SPAD units) of flag leaves from BC**_**2**_**NILs.** SPAD units of NILs carrying the Rialto (red) or Spark (blue) 6A interval in 2010 (solid lines) and 2011 (dashed lines) after anthesis. Significant differences are represented by * (*P* <0.05), ** (*P* <0.01) and *** (*P* <0.001).

Quantitative SPAD measurements were taken from the flag leaves of BC_2_ NILs in 2010 and 2011 to confirm the qualitative plot assessments and measure the rate of senescence (Figure 6). In both years, no significant differences were observed in relative chlorophyll content at 20–25 days after anthesis (DAA). Significant differences, however, were detected at 33 DAA in 2010 (*P* < 0.01) and at 62 DAA in 2011(*P* < 0.001), with Spark NILs showing significantly lower relative chlorophyll content in flag leaves compared to the Rialto NILs. Tiller number were assessed from 2011 to 2013 and again highlighted the large variation in growing seasons with numbers ranging from a mean of 76.5 tillers per m row in 2011 to 171.1 in 2012 (Table 2). Significantly fewer tillers were recorded in the higher yielding Rialto NILs in both 2012 (*P* < 0.05) and 2013 (*P* = 0.01), but not 2011. No significant correlations were observed between tiller number, yield and TGW in all three years.

### Sequence analysis of *TaGW2*

In rice, the RING-type E3 ubiquitin ligase *OsGW2* has been shown to negatively affect grain width. The wheat homologue *TaGW2* has been mapped to chromosome 6A [[17]] and was, therefore, considered as a potential candidate gene for *Qtgw-jic.6A* (and *Qyld-jic.6A*). Rialto, which produced wider grains and increased TGW and yield was found to contain the G allele at the −593 promoter SNP. Using this polymorphism, *TaGW2* was mapped on the Spark x Rialto DH population to chromosome 6A co-locating with markers *BS000072146*, *BS000105973* and *CA643341* at 46.8 cM (Figure 2), within the 8 cM peak interval determined by MTME (43 to 51 cM). Sequencing of *TaGW2* gDNA revealed only the single SNP at position −593 of the 1,244 bp upstream of the start codon between Spark and Rialto. No additional polymorphisms were detected in the A-genome coding sequence nor in the splice sites between Spark and Rialto. Likewise, no SNPs or alternative splicing variants were identified for the A-genome from Spark and Rialto cDNA samples.

## Discussion

### The interplay between TGW/yield/green canopy duration

QTL analysis of the Spark x Rialto DH population demonstrated the co-localisation of QTL determining green canopy duration, grain size (TGW) and grain yield within an 8 cM interval on chromosome 6A. These effects were confirmed with the development and assessment of independent NILs of the Rialto region into the Spark background. It was established that the extension of the green canopy duration *Qgcd-jic.6A* was comprised of two independent traits, both earlier flowering and a delay in the final senescence which resulted in a significant extension of the green canopy duration. Both component traits mapped to the Rialto 6A introgression, but their genetic relationship (pleiotropy or linkage) could not be established with the germplasm used in this study.

Green canopy duration after heading is commonly, yet mistakenly, used interchangeably with extended grain filling duration. This common generalisation leads many studies to conclude that extended green canopy duration leads to extended grain filling duration thereby resulting in larger grains and increased yield [[26],[27]]. We have shown, however, that this is not a direct/causal relationship in all cases. Caution should be exercised when interpreting the outcome of delayed leaf and plant senescence, especially in the absence of direct measurements on grain moisture content and maturity. For example, RNAi lines for the *GPC* gene confer an extended green canopy duration of >25 days [[28]], yet this is not accompanied by an extension in grain filling duration *per se* (Borrill and Uauy, unpublished results). The results of our current study also suggest that this mechanism is not the main driver behind the Rialto 6A yield effect. *Qgcd-jic.6A* showed no correlation with yield or TGW in the DH population, and despite a significant difference in GCD between NILs in 2012 (3.4 days) no yield or grain size benefit was observed. This suggests that *Qgcd-jic.6A* is most likely an independent genetic locus within the introgressed region (and not a pleiotropic effect of the yield and TGW QTL), and that extended GCD is not the major determinant behind the increase in productivity observed in the DH population and NILs.

Although the relative magnitude of effects between grain size and yield were not always consistent, a significant positive correlation was observed between the two traits across environments, suggesting that grain weight is most likely the principal yield component for *Qyld-jic.6A*. In four of the five NIL validation experiments the Rialto introgression gave significant improvements in yield (5.5 ± 1.5%) and TGW (5.1 ± 1.6%), with morphometric measurements showing that the increased grain weight was predominantly a result of wider grains. The final weight of individual grains is the last of the yield components to be established once the number of spikes per surface area and the grain number per spike have already been fixed [[4]]. This close correlation between grain weight and yield provides what could be seen as a more precise route to increased yields, compared to manipulating other yield components, as it is stably inherited [[6]] and compensatory effects are limited. Given the current rate of genetic gain in hexaploid wheat breeding, the >5% yield effect is equivalent to ~10 years of breeding, without considering additional phenotypic variation which could be identified in homoeologous loci for the 6A QTL. Ultimately, fine mapping though recombinant inbred lines will assist in determining the precise location of the traits discussed here and determine if the yield effect is pleiotropic to grain weight (and/or green canopy duration).

Compared to the large number of QTL studies for yield and other complex traits in wheat, there have been relatively few studies into individual grain morphometric components. Ramya et al. [[29]] identified several independent genetic loci for TGW and grain length and width in a large DH population, with four of these QTL suggested as pleiotropic for TGW and length/width parameters. In the current study we did not assess the original DH population for grain morphometric parameters due to the laborious nature of this task when the original field trials were conducted (using a hand-held calliper). However, with the development of high throughout phenotyping equipment, such as the Marvin Grain Analyzer, we were able to assess the NILs in greater detail and determine the most likely effect underlying the increase TGW. These new phenotyping tools should greatly facilitate the genetic dissection of grain size parameters in wheat in the coming years.

### Absence of yield and TGW effect in 2012

In the 2012 UK growing environment no effect was seen in TGW and a negative response to yield was observed in the Rialto NILs despite the otherwise consistent positive effects in the additional four environments. A possible explanation for this inconsistent result is the presence of an undesirable minor QTL for tiller number within the Rialto introgressed region that maps distal to the grain size and yield effects. We detected a minor QTL in the DH lines using the MTME analysis in three of the five environments tested, although this effect was borderline significant (LOD = 2.3, see Additional file 3). Similar effects of lower tiller number in Rialto NILs were observed in 2012 and 2013; however, they only affected the expression of the 6A yield and TGW effects in 2012. This growing season was uncharacteristic in the UK due to extreme weather contrasts. The winter of 2011 and spring of 2012 were relatively warm and dry compared to the UK historical averages, leading to high levels of tiller production; however this was followed by an exceptionally wet period from April through much of the summer. Specifically for the 6A NILs, we observed a two-fold increase in tiller number compared to the averages of the 2011 and 2013 seasons. It is plausible that the higher tiller number in 2012 led to additional genetic x environment interactions and compensatory effects in the NILs which affected the expression of the 6A TGW and yield QTL.

### *TaGW2* as a potential candidate gene for *Qyld-jic.6A* and *Qtgw-jic.6A*

The MTME QTL analysis defined an 8 cM region between *wPt-7063* (43.1 cM) and *Xgwm256* (51 cM) in which both *Qyld-jic.6A* and *Qtgw-jic.6A* co-localised. Within this region we mapped *TaGW2*, the wheat homologue of *OsGW2*. Loss of function of *OsGW2* in rice leads to increased cell numbers in the spikelet hull (palea and lemma), resulting in wider grains and indirectly affecting grain filling rates [[16]]. The combined effects lead to a 50% higher TGW in NILs with the loss of function *OsGW2* allele, and multiple other pleiotropic effects. In first instance, the similarity of effects between *Qyld-jic.6A*/*Qtgw-jic.6A* and *OsGW2* on TGW and grain width, and their syntenic positions in wheat and rice, respectively, suggests that *TaGW2* is a compelling candidate gene for *Qyld-jic.6A*/*Qtgw-jic.6A*.

There are several results, however, which suggest a cautious approach is needed in evaluating the possible role of *TaGW2* as *Qyld-jic.6A*/*Qtgw-jic.6A*. First, the effect of *Qtgw-jic.6A* on TGW is relatively small compared to that seen in rice (50%), with NILs carrying the positive effect QTL interval showing between 2 and 9% higher TGW. This could be in part due to the polyploid nature of hexaploid wheat, with the additional homoeologues resulting in a dosage effect causing the phenotype variation to be more subtle. Second, the direct effect of *OsGW2* is on palea and lemma cell number which would be less relevant in the case of wheat due to the small role that these structures play on grain width and size. Third, the allelic variation at the sequence level between the Spark and Rialto parents is limited to a sole SNP in the promoter region which has been loosely associated with TGW and width although with contradictory results [[17],[18]]. Perhaps the best evidence that *TaGW2* does indeed affect TGW and grain width in polyploid wheat comes from Yang et al. [[19]] and Bednarek et al. [[20]] who show the effect through a frame-shift mutation and RNAi, respectively. However, their results point to opposite effects for *TaGW2*. It is too early to conclude if and how *TaGW2* affects grain width and TGW in the Spark x Rialto population, but several additional experiments including detailed expression analysis and the fine mapping of *Qyld-jic.6A*/*Qtgw-jic.6A* with respect to *TaGW2* will help elucidate this relationship.

### Genetic architecture of quantitative traits in wheat

In rice, the grain size QTLs identified from bi-parental mapping populations have relatively large effects on TGW and yield, whereas in wheat the majority of studies have identified QTL with relatively small additive effects on grain size (and yield). These differences could be due to several reasons. First, the biological mechanisms determining grain size and yield might be different across species. For example, several rice QTL affect grain size indirectly through the modulation of spikelet hull and glume size [[16],[30]–[32]]. These indirect effects might be species-specific since in wheat, the palea and lemma (equivalent to the rice hull) are not so firmly attached to the grain and most likely do not determine its growth in the same manner as in rice. Second, the polyploid nature of wheat means that in many cases, mutations in individual homoeologues can be masked by functional complementation with homoeologous genes present in the other genomes. This redundancy suggests that for many genes in wheat, their full effect will only be quantifiable when all two/three homoeologues are simultaneously mutated. This problem is further confounded for quantitative traits where multiple genes contribute to a specific phenotype.

A cloned QTL for grain protein content in wheat (*GPC-B1*) exemplifies the effect of polyploidy on the relative magnitude of a quantitative trait. The original mapping of the QTL identified phenotypic variation in grain protein content between NILs of approximately 10% [[33]]. The cloning of the gene, allowed simultaneous down-regulation of the different homoeologues using RNAi, and revealed a much larger effect in grain protein content (30% reduction) than that of the B-genome homoeologue alone [[28]]. Recently, this dosage effect has been confirmed in homozygous EMS-mutants of the A and D genome homoeologues. Individual gene knockouts of the *GPC-A1* and *GPC-D1* homoeologues had a relatively small effect on grain protein content (4% and 7%, respectively), whereas the double mutant had a significantly larger reduction of 17% [[34]].

### Future directions

The validation of these QTL using NILs provides a critical first step to further fine map both traits using a series of homozygous recombinant lines. The recent advances in wheat and *Triticeae* genomics provide a robust framework from which to identify additional markers and candidate genes to aid in this process. These advances include the public release of the wheat chromosome arm survey sequence by the International Wheat Genome Sequencing Consortium (IWGSC), the development of high throughput SNP assays [[35]–[37]], exome sequencing of TILLING populations [[38],[39]], and the ongoing progress on individual chromosome arm physical maps [[40],[41]]. However, to fully exploit these genomic advances, a shift from a quantitative to a qualitative genetic locus will ultimately be required to isolate the gene(s) underlying these grain size and yield effects.

## Conclusions

In this study we identified major and stable QTL for yield, grain weight, and green canopy duration in polyploid wheat across North European environments. These effects were validated using a series of NILs which showed, on average, over 5% improvement in yield and TGW in four of the five environments tested. Our results suggest that the yield effect is driven primarily by increased grain weight due to wider grains. The lack of correlation between prolonged green canopy duration and yield/TGW argue against a direct/causal relationship between these traits within the germplasm studied. Using a promoter polymorphism, we mapped the *TaGW2* candidate gene within the QTL interval, although it is still premature to assign a specific contribution of this gene to the QTL effect. The validation of this QTL provides an important first step to advance our understanding of the genetic mechanisms regulating the complex processes governing grain size and yield in polyploid wheat.

## Methods

### Plant material

A Doubled Haploid (DH) mapping population comprising 129 individuals was developed from the cross between two UK hexaploid winter wheat cultivars (*Triticum aestivum* L.), Spark and Rialto. The cross was chosen with the aim of discovering QTL controlling yield variation in a bread making background and from independent breeding programmes. Spark, released in 1993 by Nickersons Seeds (now Limagrain), is a Group 1 premium bread making variety whereas Rialto, released in 1991 by PBIC (now RAGT), is a Group 2 bread making variety, as classified in the nabim wheat guide [[42]]. The population was created using the standard wheat x maize technique from F_1_ plants [[43]].

To independently validate the multiple QTL effects on chromosome 6A, we developed a set of near isogenic lines (NILs) using markers *Xwmc32*, *Xpsp3071* and *Xgwm570* for marker assisted backcrossing. This was accomplished by backcrossing two DH lines (56 and 136) homozygous for Rialto loci across the *Xwmc32*-*Xgwm570* interval, to the recurrent parent Spark. The lines were advanced to BC_2_ (and continued to BC_4_) by crossing heterozygous plants selected at each generation using microsatellite markers *Xwmc32*, *Xpsp3071* and *Xgwm570*. After the final backcross, selected heterozygotes were self-pollinated and NILs homozygous for the *Xwmc32*-*Xgwm570* interval were extracted from the resultant BC_2_F_2_ and BC_4_F_2_ plants. In total we generated 10 homozygous BC_2_ NILs (six with the Spark and four with the Rialto introgression) and eight BC_4_ NILs (four Spark and four Rialto).

### Genetic map construction and QTL analysis

Plant nuclear DNA isolations were performed using published protocols [[2]]. The genotyping procedures used have been described previously; PCR and PAGE [[44]], single strand conformation polymorphism (SSCP) [[45]], and KASPar [[46]]. Amplification conditions for SSR markers are available at the GrainGenes website [[47]].

The genetic map for Spark x Rialto was initially developed using publically available simple sequence repeat (SSR) markers. A total of 285 primer sets from JIC/*psp* [[44],[48]], IPK Gatersleben/*gwm/gdm* [[49],[50]], Wheat Microsatellite Consortium/*wmc* [[51]], Beltsville Agricultural Research Station/*barc* [[52]] and INRA/*cfa/cfd* [[53]] collections were polymorphic between Spark and Rialto. To ensure maximum genome coverage, 164 markers were selected for population mapping using published consensus maps [[21]] aiming for a marker density every 20 cM where possible. *Rht-D1* alleles segregated in this cross and were mapped using the ‘perfect’ markers [[54]] and alternatively with a GA seedling test. To improve map density, DNA of the population was sent to Triticarte Pty Ltd, Australia [[55]] for Diversity Arrays Technology (DArT) genome profiling [[56]] (for full genotypes see Additional file 5).

Linkage analysis and genetic map construction was performed using JoinMap® version 3.0 [[57]], using the default settings. Linkage groups were determined using a Divergent log-of-odds (LOD) threshold of 3.0 and genetic distances were computed using the Kosambi regression.

To increase marker resolution across the 6A QTL interval, an additional 9 single nucleotide polymorphism (SNP) markers derived from expressed sequence tags (EST [[58]]) were mapped using SSCP. A further 19 KASPar SNP markers [[59]] previously assigned to chromosome 6A [[35],[60]–[62]] were also screened and added to the genetic map. *TaGW2* was mapped using the cleaved amplified polymorphism sequence (CAPS) markers (Hap-6A-P1 and Hap-6A-P2) as previously described [[17]]. An additional 33 KASPar SNP markers predicted to be distal to *Xgwm570* [[59]] were monomorphic between Spark and Rialto.

QTL Cartographer v2.5 [[63]] was used for QTL detection and to estimate QTL effects using single marker analysis and the composite interval mapping (CIM) function. CIM Model 6 was selected using five control markers, a window size of 10 cM and the backward regression method. A LOD significance threshold of 2.5 was used with a 1 cM walking speed, 500 permutations and a significance level of 0.05. Estimates of additive effects and percentage of total variation for identified QTL were calculated using multiple interval mapping (MIM). This was complemented by multi-trait multi-environment (MTME) analysis [[25]] for QTL detection in Genstat 15^th^ edition (VSN International). The most suitable variance–covariance structure was detected (Factor Analytic Order 1) and genetic predictors were computed for a step size of 4 cM. The significance levels and QTL effects were determined by a final backward selection step at a significance level of 0.05.

### Field evaluation and phenotyping

The DH population was grown in a randomised complete block design with three replications at five sites (Norwich, England (52°37′39.9“N, 1°10′45.9”E); Sandringham, England (52°50′02.1“N, 0°25′48.9”E); Balmonth, Scotland (56°15′02.3“N, 2°44′31.5”W); Bohnshausen, Germany (51°51′31.8“N, 10°57′38.5”E); and Froissy, France (49°34′07.6“N, 2°13′11.6”E)). The experiments were grown over three years (2001–2003) at Norwich and Sandringham, and for two years (2002–2003) at the other three locations. Trials were sown in large-scale yield plots (1.1 × 6 m) and standard farm pesticide and fertiliser applications were made to reproduce commercial practise. Trials were sown by grain number to ensure comparable plant densities for each plot aiming for a target population of 275 seeds*m^−2^.

Final plot yield, after correction for plot size, was recorded at all sites and additional phenotypic assessments of developmental traits and yield components were made at Norwich and Sandringham. These included heading date at Zadoks growth stage 57 [[64]] and physiological maturity (GS91; measured as loss of green colour in 50% of the peduncles) (Norwich 2001–2003 and Sandringham 2001). Green canopy duration after heading was calculated by subtracting the number of days from drilling to heading, from the number of days to physiological maturity. Tiller number was assessed by counting the number of fertile tillers along a 1 m row (2 measurements per plot) (Norwich 2001–2003 and Sandringham 2002–2003). Prior to harvest, 10 main tillers (from 10 different plants) were sampled from each plot for assessments of yield components including plant biomass, harvest index, seeds/spike, seeds/spikelet and TGW (for full phenotypic scores of DH population see Additional file 6).

The NILs were grown at Norwich in 2010–2012 (10 BC_2_ NILs) and 2013 (8 BC_4_ NILs) and at Wohlde, Germany (52°48′28.5“N, 9°59′54.8”E) in 2012 (10 BC_2_ NILs). Trials were sown with the same experimental practices as the DH population, with the exception of the number of replicates (5–10 at Norwich and 3 at Wohlde). Field phenotyping was conducted in the same manner as for the DH population (described in preceding paragraph) with the following exceptions; biomass and harvest index were only measured in 2011 and tiller number, seeds/spike and seeds/spikelet were measured only in 2011 to 2013 in Norwich. Morphometric measurements (grain width, length, area and thousand grain weight) were recorded from 300–400 grains per sample using the MARVIN grain analyser (GTA Sensorik GmbH, Germany). In addition, field grown NILs were checked with DNA markers to confirm their identity both in the ground and after harvest.

In addition, the relative chlorophyll content was measured in 2010 and 2011 using a SPAD 502-Plus meter (Konica Minolta, UK). SPAD measurements were taken at several time-points after anthesis, aiming for an initial reading when there was no visible difference between the NILs, and then at suitable time points thereafter as senescence progressed. Ten main tillers per plot were tagged at anthesis and used for measurements throughout grain filling. Three measurements were taken across the flag leaf (base, middle and tip) and a mean value obtained for each tagged tiller.

### Statistical analysis

DH lines were classified according to their genotype across the *Xgdm36* to *Xgwm570* genetic interval (35 homozygous for Rialto and 49 homozygous for Spark). Based on this classification, general one-way analyses of variance (ANOVA) were performed for the multiple traits and Pearson’s correlation coefficients were calculated between selected traits. NILs were evaluated using two-way ANOVA in which the interaction between environment and the 6A interval was included in the model. ANOVA and correlations were performed using GenStat 15^th^ edition (VSN International).

### *TaGW2* sequencing

#### gDNA

Primers to amplify the genomic DNA of the A-genome homoeologue of *TaGW2* from Spark and Rialto were designed based on the International Wheat Genome Sequencing Consortium (IWGSC) Chinese Spring chromosome arm survey sequence available at URGI [[65]]. Contigs 6AS_4408273 and 6AS_4409759 (corresponding to upstream sequence and exons 1 to 6, and exons 7 and 8, respectively) were annotated based on published *TaGW2* cDNA sequence [[20]]. Four homoeologue-specific primer pairs were designed to amplify the genomic DNA encompassing 1,244 bp upstream of the ATG start codon, exon 1, exons 2 to 6, and exons 7 and 8 (Additional file 7). A touchdown PCR programme was used to amplify genomic DNA of Spark and Rialto, and PCR products were directly sequenced using a BigDye Terminator v3.1 kit (Invitrogen). Electrophoresis of products and fluorescence trace data generation were conducted by The Genome Analysis Centre (TGAC), Norwich.

#### cDNA

Total RNA was extracted from leaf sections of one 4-leaf seedling each of Spark and Rialto using Tri-Reagent (Sigma, St. Louis, USA) and cDNA was synthesized using MMLV-RT (Invitrogen, Carlsbad, USA) using the manufacturer’s protocols. Wheat ESTs and bespoke assemblies from the 454 5× raw data of hexaploid wheat Chinese Spring [[59]] were used to design primers in the untranslated regions (UTR) of *TaGW2*. Primers were designed to amplify the three homoeologous genomes. Using these primers and the cDNAs of Spark and Rialto, a RT-PCR was used to amplify the complete *TaGW2* CDS of the two varieties (all three genomes). Purified RT-PCR products were modified with A-overhangs by incubating with 10 mM dATP and 1u of Taq polymerase (Promega, Madison, USA) in 1× manufacturer’s buffer for 10 mins at 72°C. They were then cloned directly using a pGEMT-Easy Kit (Promega) following the manufacturers’ instructions. Miniprep DNA of clones was insert-sequenced using M13 primers and sequenced by TGAC. Sequence reads were aligned and assigned to the A genome using the sequence of the flow sorted chromosome arm DNA [[66]] and the IWGSC survey sequence which became publicly available afterwards [[67]].

### Availability of supporting data

The data sets supporting the results of this article are included within the article and its additional files.

## Competing interests

The authors declare that they have no competing interests.

## Authors’ contributions

JSi developed the germplasm used in this study, carried out the mapping, coordinated all field evaluations and statistical analysis and wrote the manuscript. PS provided assistance with phenotypic assessments, field trial preparation and glasshouse husbandry. MLW led the mapping of the original Spark x Rialto DH population and assisted with phenotypic assessments of the population. AT and JB carried out the sequencing of *TaGW2*. VK coordinated field trials in Germany. JSn coordinated and conceived the DH population field trials. CU conceived the NIL validation experiments, analysed the data, and wrote the manuscript. All authors read and approved the final manuscript.

## Additional files

## Supplementary Material

Additional file 1: **Yield and TGW QTL effects in DH population across individual environments.** Genome-wide QTL analyses for yield (a) and thousand grain weight (b) in the Spark x Rialto DH population. Environments are determined by colour (Norwich, blue; Sandringham, taupe; Scotland, red; France, grey; Germany, green) and years by line style (2001, solid line; 2002, large dashed line; and 2003, small dashed line). The threshold value for significance is set at 2.5 LOD. Click here for file

Additional file 2: **Effects of the Rialto 6A region on yield, TGW and GCD in DH lines across environments.** DH lines were classified according to their genotype across the *Xgdm36* to *Xgwm570* genetic interval (35 for Rialto and 49 for Spark) and the phenotypic values averaged for each location. Differences between the Rialto and Spark NIL phenotypes are presented as a percentage of the Spark NIL (yield and TGW) or as the absolute value (GCD). Click here for file

Additional file 3: **MTME output for agronomic and yield related traits.** Green canopy duration across 4 environments (a), thousand grain weight across 12 environments (b), tiller number across five environments (c), and yield across 12 environments (d). Significant markers are represented with a square, the colour represents the increasing parental allele (blue for Spark and yellow/red for Rialto) and the intensity of the colour demonstrates the significance level (dark blue and red indicates a higher significance). Click here for file

Additional file 4: **Effects of the Rialto 6A segment on additional agronomic and yield related traits.** Plant biomass, harvest index, spike length, spikelet number, spike yield, seeds/spike, and seeds/spikelet in BC_2_ and BC_4_ NILs. Significant differences are represented by *(*P* <0.05), **(*P* <0.01) and ***(*P* <0.001). Click here for file

Additional file 5: **Genotype of the Spark x Rialto DH population.** Genotype of 129 DH lines with 263 genetic markers scored for the Spark (A) or Rialto (B) allele. Click here for file

Additional file 6: **Phenotypic scores of the Spark x Rialto DH population across 12 environments.** Yield (kg/plot) and thousand grain weight (g) of the 129 DH lines grown across different environments. Click here for file

Additional file 7: **Primer sequences used to amplify** ***TaGW2-A*** **genomic DNA and cDNA.** Click here for file
