# Supplementary figures and images for: Identification and independent validation of a stable yield and thousand grain weight QTL on chromosome 6A of hexaploid wheat (Triticum aestivum L.)
Source: BMC Plant Biol. 2014 Jul 18;14:191. doi: 10.1186/s12870-014-0191-9 (PMC4105860; doi:10.1186/s12870-014-0191-9)

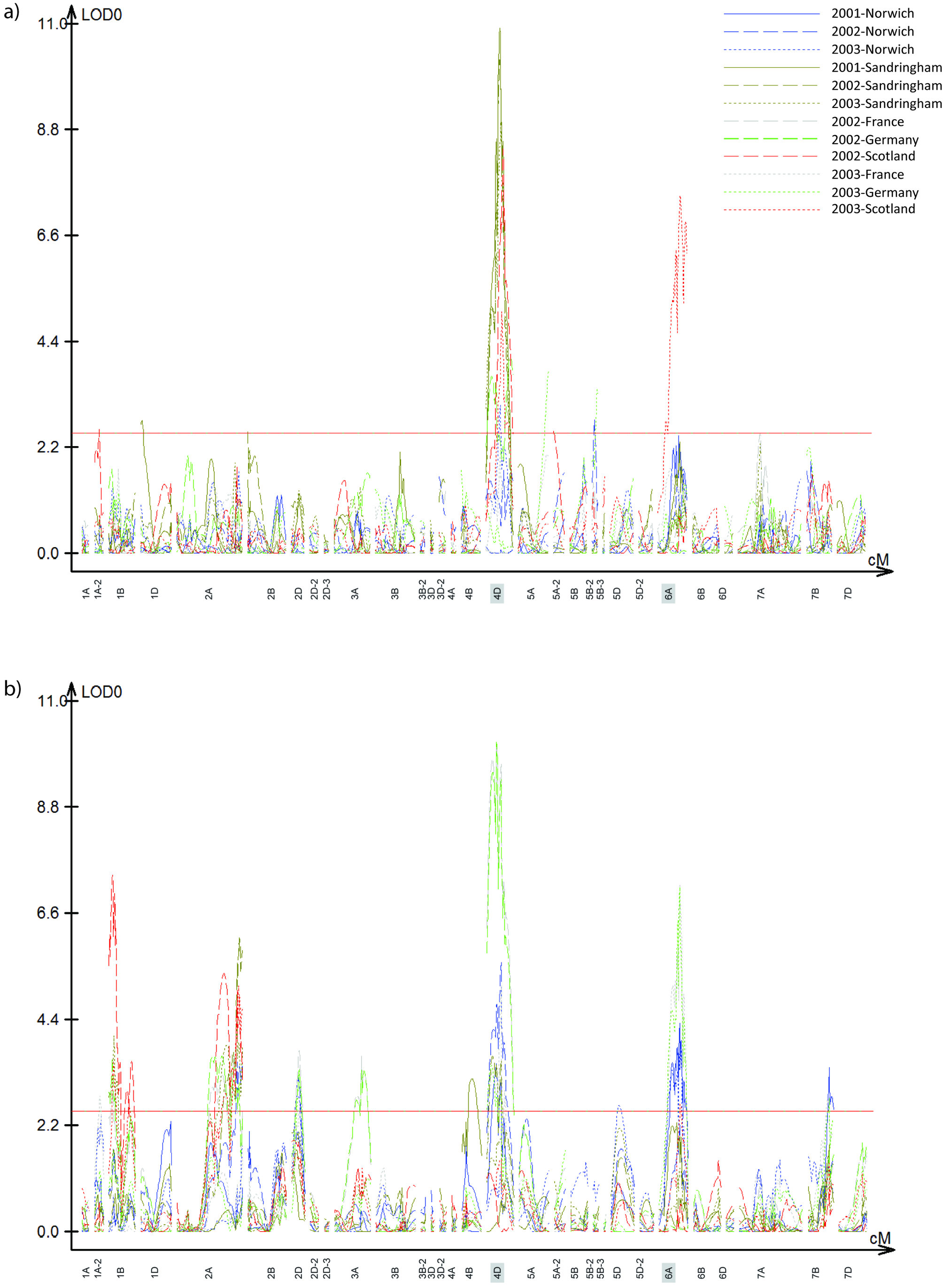

Supplement: Additional file 1: — Yield and TGW QTL effects in DH population across individual environments. Genome-wide QTL analyses for yield (a) and thousand grain weight (b) in the Spark x Rialto DH population. Environments are determined by colour (Norwich, blue; Sandringham, taupe; Scotland, red; France, grey; Germany, green) and years by line style (2001, solid line; 2002, large dashed line; and 2003, small dashed line). The threshold value for significance is set at 2.5 LOD. [file s12870-014-0191-9-S1.tiff]

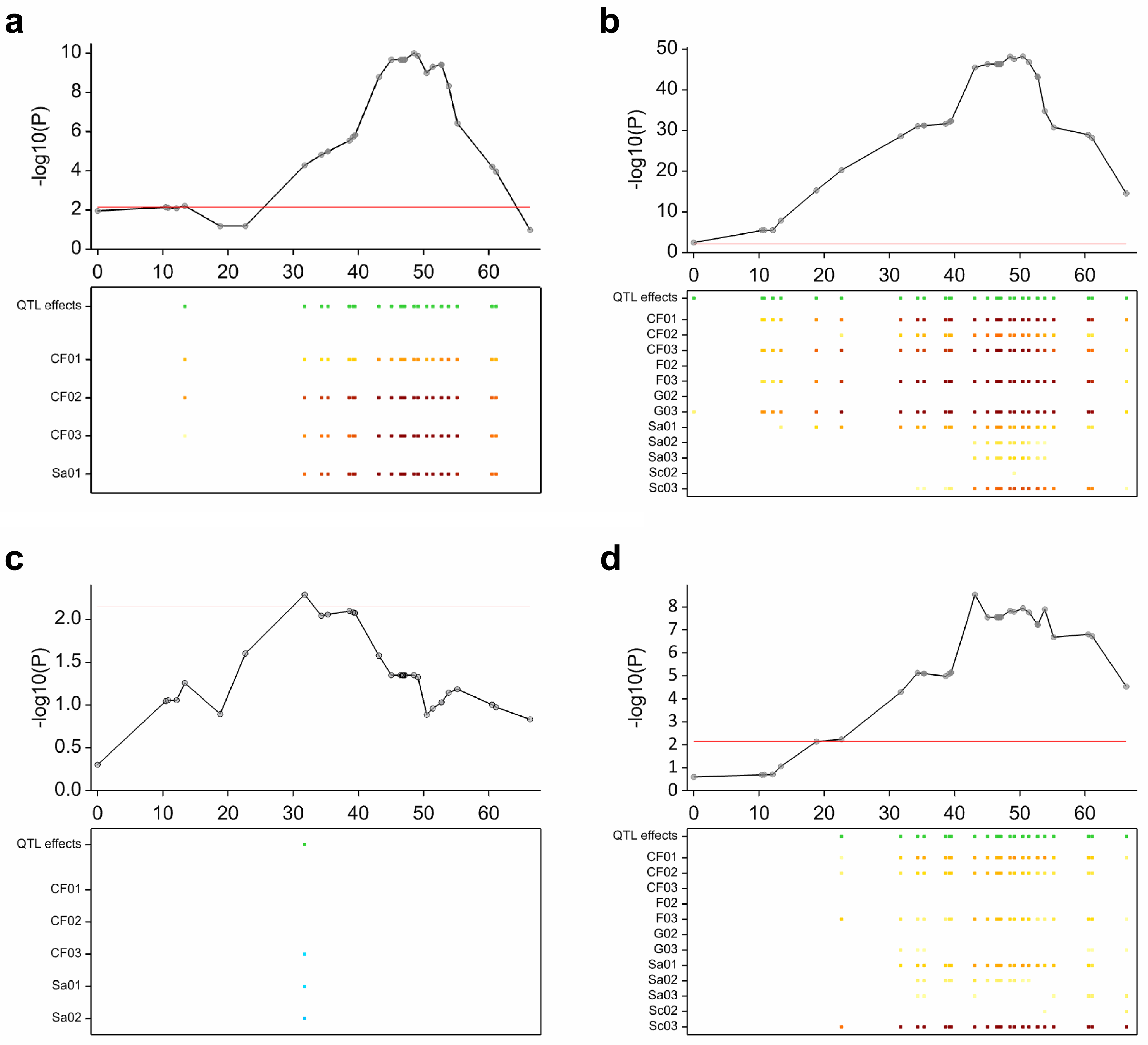

Supplement: Additional file 3: — MTME output for agronomic and yield related traits. Green canopy duration across 4 environments (a), thousand grain weight across 12 environments (b), tiller number across five environments (c), and yield across 12 environments (d). Significant markers are represented with a square, the colour represents the increasing parental allele (blue for Spark and yellow/red for Rialto) and the intensity of the colour demonstrates the significance level (dark blue and red indicates a higher significance). [file s12870-014-0191-9-S3.tiff]
